# Seroprevalence of Antibodies Specific to Receptor Binding Domain of SARS-CoV-2 and Vaccination Coverage Among Adults in Los Angeles County, April 2021: The LA Pandemic Surveillance Cohort Study

**DOI:** 10.1001/jamanetworkopen.2021.44258

**Published:** 2022-01-20

**Authors:** Neeraj Sood, Olivier Pernet, Chun Nok Lam, Angela Klipp, Rani Kotha, Andrea Kovacs, Howard Hu

**Affiliations:** 1 Sol Price School of Public Policy, University of Southern California, Los Angeles; 2 Schaeffer Center for Health Policy & Economics, University of Southern California, Los Angeles; 3 Keck School of Medicine, University of Southern California, Los Angeles

## Abstract

This cross-sectional study estimates the seroprevalence of antibodies specific to the receptor binding domain of the spike protein of SARS-CoV-2 and vaccination coverage among adults in Los Angeles County, California, in April 2021.

## Introduction

Understanding the presence of adaptive immune responses that are associated with protection from disease (potential protective immunity) caused by SARS-CoV-2 at the population level is critical for public policy. Potential protective immunity can be acquired either through vaccination or past infection.^1,2,3^ We used history of vaccination or presence of antibodies specific to the receptor binding domain (RBD antibodies) of the spike protein of the SARS-CoV-2 virus as potential markers for potential protective immunity as both are strongly associated with presence of neutralizing antibodies.^4,5^ We conducted surveys and serologic tests in a representative community sample to estimate the fraction of the Los Angeles County (LAC) adult population that had potential protective immunity in April 2021. We tested for differences in potential protective immunity by demographics and whether presence of RBD antibodies waned with time since infection.

## Methods

This cross-sectional study was approved by the LAC Department of Public Health (DPH) institutional review board and electronic informed consent was obtained. This study follows the Strengthening the Reporting of Observational Studies in Epidemiology (STROBE) reporting guideline.

We tested for RBD antibodies using an FDA emergency use authorized assay (Luminex xMAP SARS-CoV-2). We established 8 testing sites in LAC and residents who lived within a 15-mile radius (99% of LAC population) were eligible for participation. Testing was offered from April 9 to April 25, 2021. We used a proprietary database representing about a quarter of the LAC population maintained by a market research firm (LRW Group, a Material Company), to select participants. A random sample of these residents were invited, with quotas for enrollment for subgroups based on demographics of LAC residents. All participants were invited to participate in antibody testing and participants self-selected to consent for testing. Participants who had participated in a similar study conducted in April 2020 were oversampled.^6^ Weights were used to adjust results for LAC 2019 census estimates of demographics and LAC DPH data on confirmed COVID-19 cases and COVID-19 vaccination rates by race and ethnicity, age, and gender. Data on race and ethnicity were self reported by residents; race and ethnicity data were collected to document disparities in rate of potential protective immunity by race and ethnicity.

We used these weighted data to estimate the fraction of LAC adults that had received at least 1 dose of vaccine or had RBD antibodies, as a whole, and by vaccination status, demographics, and area poverty level. Statistical analysis was performed using Stata version 15 (StataCorp) from April to November 2021.

## Results

Of 5500 adults who completed a screener to determine eligibility for the study, 2314 (42%) provided consent and 1335 (57.7%) were tested. Of the 1335 tested adults, 790 (59.2%) were female, 672 (50.3%) were aged 30 to 49 years, 186 (13.9%) were Asian individuals, 125 (9.4%) were Black individuals, 463 (34.7%) were Hispanic individuals, 498 (37.3%) were non-Hispanic White individuals, and 413 (30.9%) had annual household income less than $50 000. Table 1 shows that the weighted sample characteristics matched the LAC population on demographics as well as percentage with confirmed COVID-19 diagnosis and percentage vaccinated. Of the weighted sample, 72.2% (95% CI, 69.8%-74.4%) were either vaccinated or had RBD antibodies, with Black individuals (52.5% [95% CI, 44.2%-60.7%]) and individuals from lower-income households (64.2% [95% CI, 58.5%-69.6%]) having lower rates of protection (Table 2). Of unvaccinated adults, 28.8% (95% CI, 23.3%-34.9%) had RBD antibodies, with significantly higher prevalence of RBD antibodies in poorer neighborhoods (eg, zip code poverty level ≥30%: 71.0% [95% CI, 51.8%-84.7%]). Prevalence of RBD antibodies was near universal for the fully vaccinated (99.7% [95% CI, 99.0%-99.9%]) and for the unvaccinated (97.0% [95% CI, 90.3%-99.1%]) with a self-reported prior positive COVID-19 test.

**Table 1.** Characteristics of Study Participants Compared With LA County Population Census Estimates and LA County Department of Public Health Estimates of COVID-19 Cases and Vaccinations

Characteristics	Sample size (N = 1335)	Unweighted, %	Weighted, %	LA county, adults %
Sex^a^				
Male	536	40.2	48.8	48.7
Female	790	59.2	50.6	51.3
Nonbinary	9	0.7	0.6	NA
Age, y^a^				
18-29	176	13.2	20.9	20.9
30-49	672	50.3	33.4	33.4
50-64	362	27.1	24.8	24.8
≥65	125	9.4	20.9	20.9
Race and ethnicity^a^				
Hispanic	463	34.7	45.2	45.2
Non-Hispanic				
White	498	37.3	27.6	27.9
Black	125	9.4	8.0	8.0
Asian	186	13.9	15.7	15.7
Other^b^	63	4.7	3.4	2.8
Income, annual household, $^a^				
<50 000	413	30.9	38.0	33.5
50 000-99 999	407	30.5	28.3	28.0
≥100 000	432	32.4	25.9	36.2
Prefer not to answer	83	6.2	7.8	2.3
Had positive COVID-19 test ever^c^	151	11.3	13.0	13.0
Received at least 1 dose of COVID-19 vaccine^d^	1035	77.5	60.9	61.0

Abbreviation: LA, Los Angeles.

^a^ LA County adult population estimates from IPUMS USA.

^b^ Participants in the other race and ethnicity category included those who both did not self-identify as being Hispanic and did not self identify as being (1) only White or Caucasian, or (2) only Black or African American, or (3) only Asian or Asian American.

^c^ LA County confirmed cases of COVID-19 as of April 25, 2021, from Los Angeles County Department of Public Health.

^d^ LA County number of adults vaccinated as of April 25, 2021, from Los Angeles County Department of Public Health.

**Table 2.** Weighted Proportion of Study Sample That Received at Least 1 Dose of COVID-19 Vaccine or Had Antibodies Specific to RBD Domain of SARS-CoV-2

Characteristics	Weighted proportion of entire sample with RBD antibodies or ≥1 dose of vaccine	Weighted proportion with RBD antibodies (95% CI)		
		Entire sample	Vaccinated	Unvaccinated
Entire sample	72.2 (69.8-74.4)	66.9 (64.4-69.4)	91.4 (89.5-93.0)	28.8 (23.3-34.9)
Sex				
Male	70.9 (66.6-74.9)	66.1 (61.6-70.4)	91.8 (89.0-94.0)	29.7 (20.6-40.7)
Female	73.7 (70.7-76.6)	68.1 (64.7-71.3)	91.2 (88.3-93.4)	28.3 (21.0-36.9)
Nonbinary	44.5 (14.2-79.6)	34.3 (10.0-71.0)	77.0 (36.6-95.1)	NA
Age, y				
18-29	67.2 (61.0-72.8)	61.7 (55.0-68.0)	89.6 (83.1-93.8)	30.9 (20.0-44.5)
30-39	73.2 (70.2-76.0)	66.8 (63.3-70.2)	89.8 (86.6-92.2)	29.5 (22.4-37.7)
50-64	75.7 (70.8-80.0)	68.3 (62.8-73.4)	88.7 (83.9-92.2)	30.9 (19.6-45.1)
≥65	71.4 (62.4-79.0)	70.7 (61.6-78.4)	98.8 (95.3-99.7)	22.3 (7.2-51.7)
Race and ethnicity				
Hispanic	65.9 (61.2-70.3)	63.2 (58.5-67.7)	94.4 (91.6-96.3)	33.6 (25.4-42.9)
Non-Hispanic				
White	88.6 (86.4-90.6)	79.4 (75.8-82.5)	88.9 (85.3-91.6)	31.8 (21.7-44.0)
Black	52.5 (44.2-60.7)	49.6 (41.1-58.2)	92.9 (85.0-96.8)	19.8 (9.3-37.5)
Asian	69.0 (64.6-73.1)	63.4 (58.0-68.5)	91.2 (84.9-95.0)	15.0 (6.6-30.6)
Other^a^	82.8 (71.0-90.4)	72.9 (58.3-83.8)	87.3 (67.6-95.8)	23.8 (7.3-55.3)
Income, annual household, $				
<50 000	64.2 (58.5-69.6)	60.7 (54.9-66.2)	93.1 (89.9-95.4)	27.4 (18.7-38.2)
50 000-99 999	71.8 (64.8-77.9)	65.9 (59.2-72.0)	90.5 (86.3-93.5)	25.6 (17.3-36.3)
≥100 000	81.0 (74.1-86.4)	73.6 (67.0-79.3)	90.2 (86.3-93.0)	23.5 (13.8-37.2)
Prefer not to answer	83.0 (68.4-91.7)	78.9 (64.9-88.4)	93.0 (83.5-97.3)	58.1 (32.1-80.3)
Zip code poverty level, %				
<10	72.0 (63.1-79.5)	66.9 (58.3-74.5)	92.4 (88.2-95.1)	14.5 (7.4-26.3)
10 to <20	69.9 (65.5-74.0)	64.1 (59.7-68.3)	90.5 (87.4-92.9)	21.7 (15.6-29.3)
20 to <30	72.4 (65.0-78.7)	67.1 (59.9-73.6)	90.9 (86.4-94.0)	33.6 (22.0-47.4)
30 to 100	84.9 (74.5-91.6)	83.2 (72.7-90.2)	96.4 (90.0-98.7)	71.0 (51.8-84.7)
Ever tested positive for COVID	98.4 (94.8-99.5)	98.4 (94.8-99.5)	100	97.0 (90.3-99.1)
Fully vaccinated and 2 wk after vaccination^b^	100	99.7 (98.9-99.9)	99.7 (98.9-99.9)	NA

Abbreviation: RBD, receptor binding domain.

^a^ Participants in the other race and ethnicity category included those who both did not self-identify as being Hispanic and did not self identify as being (1) only White or Caucasian, or (2) only Black or African American, or (3) only Asian or Asian American.

^b^ Two weeks after vaccination relative to study testing date.

## Discussion

This study found that in April 2021 approximately 72% of LAC adults had potential protective immunity against SARS-CoV-2. Despite this high level of population immunity and continuing vaccination efforts, LAC experienced a surge in COVID-19 cases in July 2021 suggesting that reaching herd immunity might be more difficult than anticipated,

Rates of potential protective immunity were much lower for Black individuals and those from lower-income households, which justifies increased vaccination efforts for these communities. The findings also suggest a much higher cumulative incidence of infection in high poverty areas, which likely contributed to the steep socioeconomic disparities in COVID-19 mortality rates observed in LAC.

Almost all of the unvaccinated individuals with prior infection had RBD antibodies, even though many participants had infections several months prior to antibody testing. This suggests that RBD antibodies were not waning.

The study has limitations. We used self-reported vaccinations and presence of antibodies as a marker for immunity instead of, for example, measuring cell-mediated immunity. Even though we weighted our estimates to match percentage of confirmed cases and percentage vaccinated—the 2 most important factors associated with protective immunity—we cannot rule out selection bias.
